# Supplementary material for: Intra-promoter switch of transcription initiation sites in proliferation signaling-dependent RNA metabolism
Source: Nat Struct Mol Biol. 2023 Nov 23;30(12):1970–84. doi: 10.1038/s41594-023-01156-8 (PMC10716046; doi:10.1038/s41594-023-01156-8)
Supplement: Supplementary file 1 — Reporting Summary [file 41594_2023_1156_MOESM1_ESM.pdf]

nature portfolio

Corresponding author(s): Joseph Wragg

Last updated by author(s): Oct 5, 2023

Reporting Summary

Nature Portfolio wishes to improve the reproducibility of the work that we publish. This form provides structure for consistency and transparency in reporting. For further information on Nature Portfolio policies, see our Editorial Policies and the Editorial Policy Checklist.

Statistics

For all statistical analyses, confirm that the following items are present in the figure legend, table legend, main text, or Methods section.

n/a	Confirmed	
☐	☒	The exact sample size (*n*) for each experimental group/condition, given as a discrete number and unit of measurement
☐	☒	A statement on whether measurements were taken from distinct samples or whether the same sample was measured repeatedly
☐	☒	The statistical test(s) used AND whether they are one- or two-sided<br>*Only common tests should be described solely by name; describe more complex techniques in the Methods section.*
☒	☐	A description of all covariates tested
☐	☒	A description of any assumptions or corrections, such as tests of normality and adjustment for multiple comparisons
☐	☒	A full description of the statistical parameters including central tendency (e.g. means) or other basic estimates (e.g. regression coefficient) AND variation (e.g. standard deviation) or associated estimates of uncertainty (e.g. confidence intervals)
☐	☒	For null hypothesis testing, the test statistic (e.g. *F*, *t*, *r*) with confidence intervals, effect sizes, degrees of freedom and *P* value noted<br>*Give P values as exact values whenever suitable.*
☒	☐	For Bayesian analysis, information on the choice of priors and Markov chain Monte Carlo settings
☐	☒	For hierarchical and complex designs, identification of the appropriate level for tests and full reporting of outcomes
☐	☒	Estimates of effect sizes (e.g. Cohen's *d*, Pearson's *r*), indicating how they were calculated

Our web collection on statistics for biologists contains articles on many of the points above.

Software and code

Policy information about availability of computer code

Data collection	N/A
Data analysis	Data was analyzed using publicly available software, which are cited in the paper. The version of software and syntax used are listed in the paper.

For manuscripts utilizing custom algorithms or software that are central to the research but not yet described in published literature, software must be made available to editors and reviewers. We strongly encourage code deposition in a community repository (e.g. GitHub). See the Nature Portfolio guidelines for submitting code & software for further information.

Data

Policy information about availability of data

All manuscripts must include a data availability statement. This statement should provide the following information, where applicable:

- Accession codes, unique identifiers, or web links for publicly available datasets
- A description of any restrictions on data availability
- For clinical datasets or third party data, please ensure that the statement adheres to our policy

All relevant data and results included in this article have been published along with the article and its supplementary information files. Raw sequencing data for CAGE-seq is publicly available at NCBI Sequence Read Archive under accession number PRJNA934878. Public data from FANTOM5, was downloaded from https://fantom.gsc.riken.jp/5/datafiles/latest/basic/.

Human research participants

Policy information about studies involving human research participants and Sex and Gender in Research.

Reporting on sex and gender	Sex/gender information, where available, is provided in Supplementary table 2. As described in the paper it was assessed, but not found to correlate with transcription initiation behavior. Sex/gender information was not collected under the ethics for the organoid or clinical samples, and therefore was not considered.
Population characteristics	Population characteristics are provided in Supplementary tables 2 and 4. All available characteristics were assessed with their relevance discussed in the manuscript.
Recruitment	No human participants were recruited for this study as it is pre-clinical in nature, with only anonymised archived tissues or established cultures used.
Ethics oversight	Project approval code 17-287, Human Biomaterials Resource Centre, Birmingham, United Kingdom; under ethical approval from Northwest - Haydock Research Ethics Committee (Reference: 15/NW/0079).

Note that full information on the approval of the study protocol must also be provided in the manuscript.

Field-specific reporting

Please select the one below that is the best fit for your research. If you are not sure, read the appropriate sections before making your selection.

☒ Life sciences ☐ Behavioural & social sciences ☐ Ecological, evolutionary & environmental sciences

For a reference copy of the document with all sections, see nature.com/documents/nr-reporting-summary-flat.pdf

Life sciences study design

All studies must disclose on these points even when the disclosure is negative.

Sample size	No statistical method was used to predetermine sample size as this was rather determined by maximum sample availability which was limited in the case of organoids by numbers successfully generated, clinical samples by ethics agreements and previously published FANTOM5 CAGE data by cancer samples having suitable match healthy tissue samples.
Data exclusions	No data were excluded from the analyses.
Replication	Experimental data was the product of 3 independent experiments unless otherwise stated.
Randomization	The experiments were not randomized as this this was not relevant to the study. All analyses were objective in nature and not the product of a survey or subjective scoring approach.
Blinding	Investigators were not blinded to allocation during experiments and outcome assessment as this this was not relevant to the study. All analyses were objective in nature and not the product of a survey or subjective scoring approach. The only subjective analysis was the organoid morphology scoring in figure 3h, but with a sample size of 5, blinding was not practicable and images of each organoid are shown for the reader to make their own judgment.

Reporting for specific materials, systems and methods

We require information from authors about some types of materials, experimental systems and methods used in many studies. Here, indicate whether each material, system or method listed is relevant to your study. If you are not sure if a list item applies to your research, read the appropriate section before selecting a response.

Materials & experimental systems

n/a	Involved in the study	
☒	☐	Antibodies
☒	☐	Eukaryotic cell lines
☒	☐	Palaeontology and archaeology
☒	☐	Animals and other organisms
☒	☐	Clinical data
☒	☐	Dual use research of concern

Methods

n/a	Involved in the study	
☒	☐	ChIP-seq
☒	☐	Flow cytometry
☒	☐	MRI-based neuroimaging
